# Surgical vs. nonsurgical treatment of extra-articular scapula fractures: a systematic review and meta-analysis

**DOI:** 10.1016/j.xrrt.2026.100666

**Published:** 2026-01-21

**Authors:** Lara Stehling, Elena Ricker, Lisa Klute, Leopold Henßler, Helge Knüttel, Florian Zeman, Volker Alt, Maximilian Kerschbaum

**Affiliations:** a Department of Trauma Surgery, University Medical Center Regensburg, Regensburg, Germany; b Medical Branch Library, University Library, University of Regensburg, Regensburg, Germany; c Center for Clinical Studies, University Medical Center Regensburg, Regensburg, Germany

**Keywords:** Trauma, Shoulder, Scapula, Scapula neck, Scapula body, Fracture, Floating shoulder

## Abstract

**Background:**

Scapula fractures are historically managed conservatively. Although surgical treatment has become increasingly common and is associated with favorable outcomes, comparative studies of surgical vs. nonsurgical management remain scarce. To the best of our knowledge, this meta-analysis is the first systematic comparison of outcomes in extra-articular scapula fractures according to fracture localization.

**Methods:**

MEDLINE, Embase, Cochrane Central Register of Controlled Trials, ClinicalTrials.gov, and the World Health Organization International Clinical Trials Registry Platform were systematically searched in April 2024 for studies on extra-articular scapula fractures. Two reviewers independently conducted a two-stage screening process. Patients were grouped into scapular neck, scapular body, and floating shoulder fractures, each stratified by surgical or nonsurgical management. Surgically treated floating shoulder injuries were further categorized by clavicle fixation alone or combined scapula–clavicle fixation. Outcomes included the Constant Score (CS), University of California Los Angeles Shoulder score, and Disabilities of the Arm, Shoulder, and Hand score. A random-effects meta-analysis was performed.

**Results:**

Twenty-six studies including 601 patients met the inclusion criteria. No statistical difference was observed for scapular neck fractures (*P* = .62; mean CS: surgical 93.6 vs. nonsurgical 89.6). In scapular body fractures, CS differences were not significant, while Disabilities of the Arm, Shoulder, and Hand scores showed a trend favoring surgery (*P* = .05; surgical 5.9 vs. nonsurgical 12.8). For floating shoulder injuries, CSs were similar between nonsurgical management (77.3) and clavicle fixation alone (76.7), whereas combined scapula–clavicle fixation yielded higher scores (87.0; *P* = .14).

**Conclusion:**

Surgical intervention for extra-articular scapular fractures showed no significant overall benefit, though floating shoulder injuries trended toward clinically meaningful improvement. Current evidence is limited by heterogeneity, highlighting the need for high-quality prospective studies to guide optimal management.

Scapular fractures account for 3%-5% of upper extremity fractures and approximately 1% of all fractures.^22^ Data from the U.S. National Trauma Data Bank indicate that the incidence of scapular fractures increased from 1% to 2.2% over the course of a decade. This upward trend is likely influenced by the broader use of computed tomography in trauma assessment, which has enhanced the detection of these fractures.^7^ They are frequently associated with high-energy trauma and severe concomitant injuries and have historically been managed conservatively.^8^ However, with rising survival rates following high-energy trauma, continued technological advances, and accumulating evidence of favorable postoperative outcomes, surgical management is increasingly being considered for highly displaced scapular neck and body fractures.^38^

Despite this shift, consensus regarding the optimal management of extra-articular scapula fractures remains elusive. Comparative evidence on surgical vs. nonsurgical outcomes is limited, and variability in classification systems, outcome measures, and follow-up intervals further hinders data synthesis. Most prior studies have either compared intra- vs. extra-articular fractures or examined scapular fracture treatments broadly, without stratifying results by fracture location. To address this gap, we conducted a systematic review and meta-analysis to compare surgical and nonsurgical outcomes for scapular neck fractures, scapular body fractures, and floating shoulder injuries. By evaluating these fracture types separately, we aim to provide a location-specific overview of the reported outcomes, while acknowledging that the current evidence base is insufficient to support firm clinical recommendations. This review consolidates the existing data and highlights critical knowledge gaps as well as methodological inconsistencies that limit current clinical recommendations. Ultimately, our analysis seeks to clarify the landscape of available evidence and to guide the design of future high-quality studies needed to strengthen the foundation for evidence-based decision-making and optimize care for patients with extra-articular scapular fractures. To our knowledge, this is the first comprehensive meta-analysis to provide a fracture-specific assessment of surgical vs. nonsurgical management for these injuries.

## Material and methods

This systematic review is reported according to the Preferred Reporting Items for Systematic Reviews and Meta-Analyses (PRISMA) 2020, PRISMA-S, and Terminology, Application, and Reporting of Citation Searching (TARCiS) guidelines.^20^^,^^28^^,^^30^ The protocol was prospectively registered in International Prospective Register of Systematic Reviews Center for Reviews and Dissemination (ID: 42024549695).

### Eligibility criteria

Eligibility criteria were defined in accordance with our Population, Intervention, Comparison, Outcome framework, specifying the population, interventions, comparisons, and functional outcomes of interest.

Due to the limited number of randomized controlled trials (RCTs) directly comparing surgical and nonsurgical interventions, we included RCTs evaluating either treatment modality individually, as well as non-RCTs, prospective cohort studies, and retrospective analyses. Reviews, editorials, letters, expert opinions, animal studies, biomechanical investigations, and cadaveric studies were excluded. Only full-text articles published in English and involving patients aged 18 years and older were eligible. A minimum follow-up duration of 12 months was required. Eligibility was independent of publication date and sample size.

Inclusion criteria compromised extra-articular scapular body and neck fractures treated surgically or nonsurgically, and floating shoulder injuries managed nonsurgically, by clavicular fixation alone, or by combined scapular and clavicular fixation. No restrictions were imposed on the timing of intervention. Where multiple follow-up intervals were reported, the latest was extracted. Functional outcomes were included only when assessed with Constant Score (CS), Disabilities of the Arm, Shoulder, and Hand (DASH), or University of California Los Angeles (UCLA) score.

Exclusion criteria were studies of scapula process or intra-articular fractures and studies lacking fracture-specific outcome reporting. Fracture entities were differentiated; however, the surgical cohort encompassed diverse osteosynthesis techniques that could not be analyzed separately.

### Searching

We searched in April 2024 the following databases and registers for completed and ongoing studies using a search strategy with a single concept “population: scapula fractures,” aiming at high sensitivity and no limits.

• MEDLINE (Ovid)
• Embase (Ovid)
• Cochrane Central Register of Controlled Trials (Cochrane Library, Wiley)
• ClinicalTrials.gov
• World Health Organization International Clinical Trials Registry Platform search portal (http://apps.who.int/trialsearch/)

In addition to the database searches, we performed a backward citation search by reviewing the reference lists of the included studies using Lens.org with the citation-chaser Shiny app.^17^ References not indexed in Lens.org were screened manually.

Complete and reproducible search strategies for all databases and the citation search, accession numbers of the records found, deduplication processes, as well as PRISMA-S and TARCiS checklists for the search process are provided in the supporting data, which have been deposited in a public repository (http://doi.org/10.5283/epub.78494).

### Selection of studies

Two reviews (LS, ER) independently performed an initial screening phase of titles and abstracts using Covidence^39^ to identify potentially eligible studies. Each reviewer worked separately, and all records were screened against the prespecified eligibility criteria within the platform. At this stage, records that clearly did not meet inclusion criteria were excluded, while those deemed potentially relevant by either reviewer were carried forward for further assessment. The platform automatically generated lists of included and excluded studies, which were then cross-checked between reviewers to confirm accuracy.

**Figure 1:**
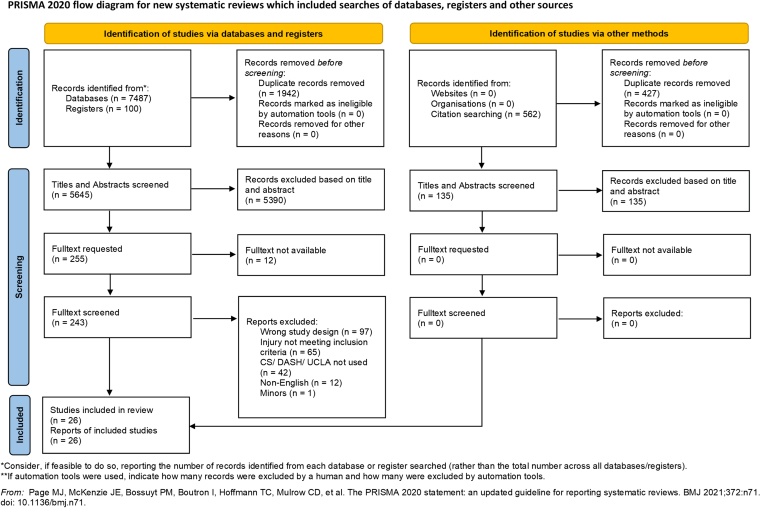
PRISMA flowchart. *PRISMA*, Preferred Reporting Items for Systematic Reviews and Meta-Analyses; *CS*, Constant Score; *UCLA*, University of California Los Angeles Shoulder score; *DASH*, Disabilities of the Arm, Shoulder, and Hand.

Full-texts were subsequently obtained for all studies that passed the title and abstract stage. These full-texts were uploaded into Covidence and reviewed in detail by both reviewers. Each study was assessed independently, with reviewers recording their decisions directly within the platform. In instances where the reviewers arrived at different decisions, the study was flagged by the system and then re-evaluated together. Discrepancies regarding the study inclusion were first addressed through discussion between the 2 reviewers. In cases where disagreements persisted due to differing interpretations, a third reviewer served as an arbitrator to reach a final decision. The study selection process is presented in Fig. 1.

**Figure 2:**
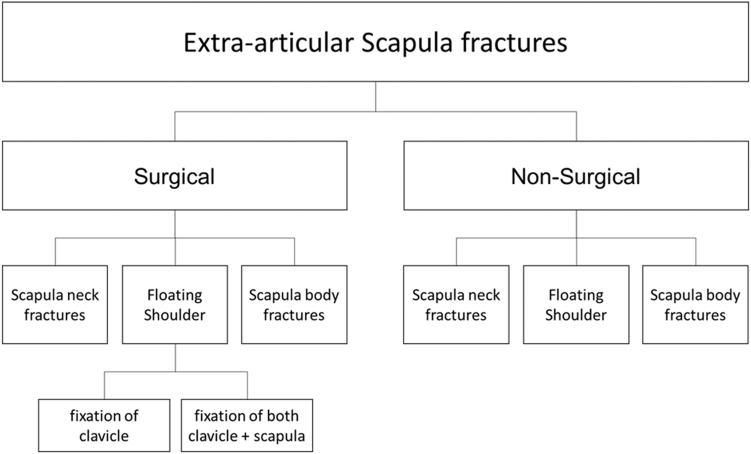
Flowchart research question.

For all studies that met the eligibility criteria, the 2 reviewers independently extracted detailed study-level and patient-level data from a total of 601 patients across 26 studies.^1^^,^^3^, ^4^, ^5^, ^6^^,^^9^, ^10^, ^11^, ^12^, ^13^^,^^16^^,^^18^^,^^19^^,^^21^^,^^23^, ^24^, ^25^^,^^27^^,^^31^, ^32^, ^33^, ^34^^,^^36^^,^^37^^,^^40^^,^^41^ Extracted variables included author, year of publication, study population, length of follow-up, and treatment modality. At the patient level, functional outcomes were extracted and limited to the CS, DASH score, and UCLA score, when these measures were reported. Special attention was given during extraction on whether the intervention involved surgical or nonsurgical management and to the type of fracture included in each study. This information was documented systematically to allow subgroup analyses. Fig. 2 illustrates the articulation of our research question in relation to the subgroups defined for the study. All data were systematically recorded and managed using Microsoft Excel (Redmond, WA, USA).

### Methodological quality assessment

The methodological quality assessment was evaluated using the Risk of Bias in Non-Randomized Studies of Interventions tool^35^ developed by the Cochrane Collaboration. The assessment addressed the following domains of bias: confounding, selection of participants, classification of interventions, deviations from intended interventions, missing data, measurement of outcomes, and selection of the reported result. Each study was assessed independently by 2 reviewers, with judgments recorded for every domain as “low risk,” “moderate risk,” “high risk,” or “no information.” To enhance the clarity and accessibility of the risk-of-bias evaluation, the risk-of-bias visualization tool^26^ was employed. This software enabled the creation of traffic-light plots for domain-specific assessments as well as weighted bar plots summarizing the overall distribution of risk-of-bias judgments across all the included studies. These visual outputs were used in combination with the narrative assessments to provide a comprehensive overview of the methodological quality of the evidence base.

### Statistical analysis

The statistical analysis was performed by L.S. using R Version 4.4.2 (Posit, Boston, MA, USA)^2^^,^^14^^,^^15^^,^^29^ to generate forest plots of each subgroup. Continuous outcomes were analyzed, and results are presented as mean scores for the CS, DASH, and UCLA instruments. All comparisons were accompanied by 95% confidence intervals (CIs). Heterogeneity was assessed using the Higgins I^2^ statistic and interpreted as follows: 0%-40% low, 30%-60% moderate, 50%-90% substantial, and 75%-100% considerable heterogeneity. Forest plots were used to illustrate the results of the analysis and to summarize effect estimates. Statistical significance was defined at a *P* value threshold of <.05. For outcomes with insufficient data to perform meta-analysis, results were reported descriptively.

## Results

### Study identification

The database search identified 7,587 records. After removal of duplicates, 5,645 titles and abstracts were screened, resulting in 255 articles selected for full-text review. Twelve articles could not be retrieved despite repeated author contact attempts, leaving 243 full-text reports for assessment. Of these, 217 were excluded for the following reasons: wrong study design (n = 97), injury not meeting inclusion criteria (n = 65), CS/DASH/UCLA not used (n = 42), non-English (n = 12), and inclusion of minors (n = 1). Twenty-six studies fulfilled the inclusion criteria and were included in the meta-analysis.

Backward citation search identified 562 additional records; after deduplication, 135 remained. Screening these 135 records yielded no new findings, and all were excluded. The final evidence base compromised 14 retrospective case series, 9 retrospective cohort studies, 1 prospective case series, 1 prospective cohort study, and 1 RCT.

### Methodological quality assessment

The majority of included studies were observational in nature, compromising either case series or cohort designs. These study types inherently lack randomized comparison groups, and as such, they carry intrinsic limitations with respect to internal validity and susceptibility to certain forms of bias. Across the included studies, the principal concerns identified pertained to bias in the selection of the reported result, bias due to missing data, and bias arising from potential confounding factors that were not controlled for within the study design. By contrast, the risk of bias in participant selection was generally low. Cases were consistently identified based on the presence of extra-articular scapular fractures, and the primary objective of most studies was to provide descriptive clinical insight rather than to establish causal inference or population-level representativeness. Consequently, the selection process for study participants was largely systematic and clearly reported, minimizing this particular source of bias. Similarly, the risk of bias related to intervention classification and outcome measurement were judged to be minimal.

**Figure 3:**
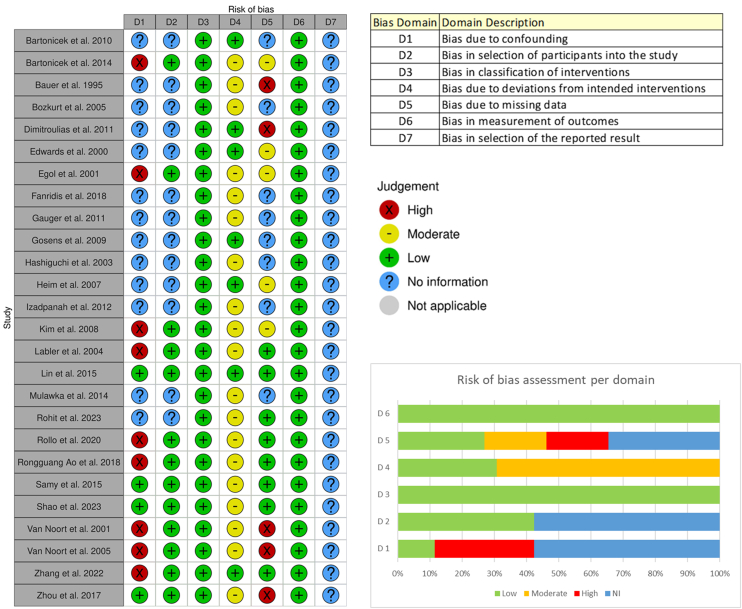
Risk of bias. *NI*, no information.

Overall, the observational design of the majority of studies imposed certain limitations. The identified areas of concern highlight the need for cautious interpretation. The results of the methodological quality assessment are presented in Fig. 3.

### Study characteristics

**Figure 4:**
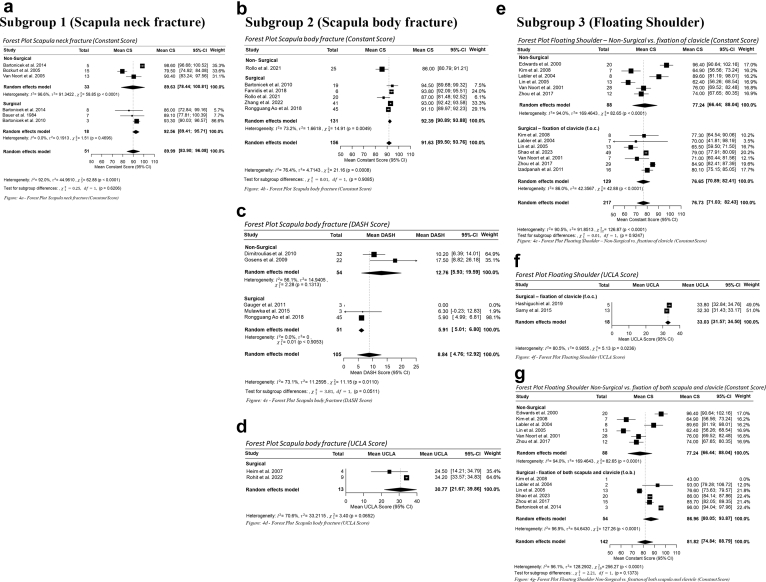
Forest plots. (**a**) Forest plot scapula neck fracture (Constant Score). (**b**) Forest plot scapula body fracture (Constant Score). (**c**) Forest plot scapula body fracture (DASH score). (**d**) Forest plot scapula body fracture (UCLA score). (**e**) Forest plot floating shoulder: nonsurgical vs. fixation of clavicle (Constant Score). (**f**) Forest plot floating shoulder (UCLA score). (**g**) Forest plot floating shoulder: nonsurgical vs. fixation of both scapula and clavicle (Constant Score). *CS*, Constant Score; *UCLA*, University of California Los Angeles Shoulder score; *DASH*, Disabilities of the Arm, Shoulder, and Hand; *CI*, confidence interval.

Data from 601 patients across 26 studies were included in the analysis. Forty-five patients were assessed with both the CS and the DASH score. Patients were stratified into 3 subgroups based on fracture location: scapular body fractures, scapular neck fractures, and floating shoulder injuries. Overall, 35% underwent nonsurgical management and 65% received surgical treatment, though this distribution varied by injury type. For scapular body fractures, the treatment proportions mirrored the overall cohort (34% nonsurgically, 66% surgically). Scapular neck fractures were more frequently managed nonsurgically (52%), whereas floating shoulder injuries were predominantly treated surgically, with only 32% managed nonsurgically. Among surgically treated floating shoulder injuries, 20% underwent fixation of both the scapula and clavicle, while 48% received fixation of the clavicle alone. The mean duration of follow-up was 30 months. The meta-analytic results are depicted in the forest plots shown in Fig. 4.

### Subgroup 1 (scapula neck fracture)

A total of 64 patients with scapular neck fractures were included in this subgroup, of whom 31 underwent surgical management and 33 were treated nonsurgically. The mean duration of follow-up was comparable between the groups, with 45 months in the surgical cohort and 46 months in the nonsurgical cohort.

CS outcomes were available for 51 patients (33 nonsurgical, 18 surgical). The mean CS was 89.6 in the nonsurgical group (95% CI, 78.4-100.8) and 92.6 in the surgical group (CI, 89.4-95.7). The difference between the 2 groups did not reach statistical significance (*P* = .62). Considerable heterogeneity was observed among the nonsurgical patients (I^2^ = 96.6%), indicating variability in outcomes across the included studies.

Additional functional outcomes from studies not included in the forest plot are presented separately. Heim et al^19^ assessed 9 surgically treated patients using the UCLA score, reporting a mean of 30.0 (range 27.6-32.4).Mulawka et al^27^ evaluated 4 surgically treated patients with the DASH score, yielding a mean of 17.5 (95% CI, 2.7-32.3).

### Subgroup 2 (scapula body fracture)

Among 229 patients with scapular body fractures, 150 underwent surgical treatment and 79 received nonsurgical management. The mean follow-up was 17 months for the surgical cohort and 27 months for the nonsurgical cohort.

CS outcomes were available for 156 patients, including 131 surgical and 25 nonsurgical cases. The mean CS was 92.4 in the surgical group (95% CI, 90.9-93.9) and 86.0 in the nonsurgical group (95% CI, 80.8-91.2). Despite this numerical difference, the comparison did not reach statistical significance (*P* = .91).

DASH scores were reported for 105 patients, compromising 51 surgically treated and 54 nonsurgically managed individuals. Of the 51 surgical patients, 45 overlap with the 131 surgical patients reported above for the CS, reflecting that these individuals were assessed with both outcome measures. The mean DASH score was 5.9 (surgical) vs. 12.8 (nonsurgical). The *P* value of .05 approaches the conventional threshold for statistical significance, suggesting a tendency toward slightly better patient-reported functional outcomes following surgical management. Thirteen surgically treated patients were assessed using the UCLA score, yielding a mean of 30.8 (95% CI, 21.7-39.9).

### Subgroup 3 (floating shoulder)

This subgroup compromised 308 patients diagnosed with floating shoulder injuries. Of these, 147 underwent open reduction and internal fixation of the clavicle alone with a mean follow-up of 24 months. Sixty-one patients underwent open reduction and internal fixation of both clavicle and scapula (mean follow-up, 23 months), and 100 were managed nonsurgically (mean follow-up, 33 months).

CS outcomes were available for 217 patients. Comparing nonsurgical management with fixation of the clavicle alone (n = 88 vs. 129), mean scores were 77.2 (95% CI, 66.4-88.0) and 76.7 (95% CI, 70.9-82.4), respectively (*P* = .92). Both cohorts exhibited wide CIs and substantial heterogeneity.

When comparing nonsurgical management (n = 88) with fixation of both scapula and clavicle (n = 54), mean CS values were 77.2 (95% CI, 66.4-88.0) and 87.0 (95% CI, 80.1-93.9), respectively, with a *P* value of .14.

UCLA scores were reported for 18 patients undergoing clavicle-only fixation, yielding a mean of 33.0 (95% CI, 31.6-34.5). DASH outcomes were reported by Egol et al^11^ for 12 nonsurgical patients (mean, 53; 95% CI, 38.4-67.6) and 7 patients received combined fixation (mean 46; 95% CI: 37.2-54.8). UCLA scores were reported for 18 patients undergoing clavicle-only fixation, yielding a mean of 33.0 (95% CI, 31.6-34.5). Meta-analysis is shown in Fig. 4 *A*a–*G*forest plots.

## Discussion

The key findings are as follows:

• The overall comparative evidence remains limited, precluding definitive treatment recommendations and highlighting the need for further high-quality studies to clarify optimal management strategies.
• No statistically significant differences were observed between surgical and nonsurgical treatment approaches.
• In patients with floating shoulder injuries, mean differences in outcomes were most pronounced and approached the threshold of the minimal clinically important difference (MCID), suggesting a potential clinical advantage of surgical management in this subgroup.

Historically, scapular fractures were predominantly managed nonsurgically. Zlowodzki et al^42^ reported that 70% of 465 cases were treated nonsurgically, with nearly all isolated body fractures managed without surgery.

Our analysis did not reveal statistically significant overall benefit of surgical vs. nonsurgical treatment. Among the different fracture types, only scapular body fractures showed a single outcome approaching statistical significance: the mean DASH score was lower in surgically managed patients (5.9) compared with those treated nonsurgically (12.8). Although this difference approached conventional statistical significance (*P* = .05), it remained below the MCID, precluding any conclusions regarding the superiority of either treatment approach.

Floating shoulder injuries, however, emerged as a notable exception. While the differences did not reach statistical significance, mean outcome scores approached the MCID (CS: 77 for nonsurgical vs. 86 for fixation of both scapula and clavicle), suggesting a potential clinically relevant superiority of combined scapular and clavicular fixation. Floating shoulder injuries are inherently unstable.^18^ The anteromedial traction of the arm on the glenohumeral joint destabilizes the anatomy, predisposing to nonunion, malunion, impingement, and degenerative joint disease.^24^ According to Egol et al^11^ neglect may result in limb shortening, persistent weakness, and chronic brachial plexopathy.

Against this backdrop, the present review synthesizes the current body of evidence, emphasizing an under-recognized injury pattern and the need for additional research. While not conclusive, the observed trends suggest that scapula fractures represent a fracture pattern meriting focused investigation. These observations underscore a critical gap in the literature and the need for prospective studies to evaluate the impact of surgery on functional outcomes and to guide evidence-based strategies for managing extra-articular scapula fractures.

### Limitations

The limitations of our meta-analysis reflect the current state of the literature on extra-articular scapula fractures. Most included studies were retrospectively designed, with only a minority being prospective, resulting in variable methodological quality and limiting the strength of causal inferences. Study designs ranged from observational cohorts to prospective series, and fracture types were not always uniformly classified, introducing additional heterogeneity. Especially the classification of floating shoulder injuries is inconsistent in literature, while some studies defining it as scapular neck plus clavicle fracture, and others including any double disruption of the superior shoulder suspensory complex. In our analysis, we included all reported floating shoulder injuries defined as a combination of scapula and clavicle fractures.

Heterogeneity was also evident in outcome assessment tools and follow-up intervals, which ranged from 12 to 74 months. This variability, combined with differing scoring systems, constrained the comparability of results and precluded more granular meta-analytic pooling for certain outcomes. Many studies reported only mean values for entire cohorts without providing individual patient-level data, limiting the ability to perform subgroup analyses or adjust for confounding factors.

The surgical group encompassed a heterogeneous array of osteosynthesis techniques, including variations in fixation methods and plate configurations, which could not be analyzed separately. Additional heterogeneity stems from variation in surgical indications, as no standardized criterion-based thresholds (eg, angulation or displacement) were consistently applied to guide surgical management. Furthermore, only a limited number of studies performed direct comparisons between surgical and nonsurgical management, restricting statistical power to detect differences.

Potential biases further limit the interpretability and generalizability of our findings. These include selection bias, reporting bias, confounding, bias due to missing data, and the risk of publication bias, which is particularly relevant in surgical literature.

Despite these limitations, pooling data from 26 selected studies provides a comprehensive overview of the current evidence and identifies overarching trends that can inform clinical decision-making and guide future research.

## Conclusion

Our analysis did not demonstrate a statistically significant overall benefit of surgical intervention for extra-articular scapular fractures, precluding definitive conclusions. Nevertheless, floating shoulder injuries exhibited a tendency toward clinically meaningful improvement, with combined clavicle and scapula fixation approaching the MCID. Although these findings are not conclusive, they underscore the limitations of the current evidence and the impact of heterogeneous outcome measures. High-quality, prospective studies are needed to clarify optimal management strategies and inform evidence-based treatment of scapula fracture.

## Disclaimers:

Funding: No external funding was received for this study.

Conflicts of interest: The authors, their immediate families, and any research foundation with which they are affiliated have not received any financial payments or other benefits from any commercial entity related to the subject of this article.

## References

[1] Ao R., Yu B., Zhu Y., Jiang X., Shi J., Zhou J. (2018). Single lateral versus medial and lateral plates for treating displaced scapular body fractures: a retrospective comparative study. J Shoulder Elbow Surg.

[2] Balduzzi S., Rücker G., Schwarzer G. (2019). How to perform a meta-analysis with R: a practical tutorial. Evid Based Ment Health.

[3] Bartoníček J., Frič V. (2011). Scapular body fractures: results of operative treatment. Int Orthop.

[4] Bartoníček J., Tuček M., Frič V., Obruba P. (2014). Fractures of the scapular neck: diagnosis, classifications and treatment. Int Orthop.

[5] Bauer G., Fleischmann W., Dussler E. (1995). Displaced scapular fractures: indication and long-term results of open reduction and internal fixation. Arch Orthop Trauma Surg.

[6] Bozkurt M., Can F., Kirdemir V., Erden Z., Demirkale I., Başbozkurt M. (2005). Conservative treatment of scapular neck fracture: the effect of stability and glenopolar angle on clinical outcome. Injury.

[7] Daher M., Abi Farraj S., El Hassan B. (2023). Management of extra-articular scapular fractures: a narrative review and proposal of a treatment algorithm. Clin Orthop Surg.

[8] Dienstknecht T., Horst K., Pishnamaz M., Sellei R.M., Kobbe P., Berner A. (2013). A meta-analysis of operative versus nonoperative treatment in 463 scapular neck fractures. Scand J Surg.

[9] Dimitroulias A., Molinero K.G., Krenk D.E., Muffly M.T., Altman D.T., Altman G.T. (2011). Outcomes of nonoperatively treated displaced scapular body fractures. Clin Orthop Relat Res.

[10] Edwards S.G., Whittle A.P., Wood G.W. (2000). Nonoperative treatment of ipsilateral fractures of the scapula and clavicle. J Bone Joint Surg Am.

[11] Egol K.A., Connor P.M., Karunakar M.A., Sims S.H., Bosse M.J., Kellam J.F. (2001). The floating shoulder: clinical and functional results. J Bone Joint Surg Am.

[12] Fandridis E., Anastasopoulos P.P., Alexiadis G., Nomikarios D., Spyridonos S., Hertel R. (2018). Posterior subdeltoid and external rotators preserving approach for reduction and fixation of displaced extra-articular fractures of the scapula. Eur J Orthop Surg Traumatol.

[13] Gauger E.M., Cole P.A. (2011). Surgical technique: a minimally invasive approach to scapula neck and body fractures. Clin Orthop Relat Res.

[14] Gohel D., Moog S., Heckmann M. (2024). Officer: manipulation of Microsoft Word and PowerPoint documents. Version 0.6.7. https://davidgohel.github.io/officer/.

[15] Gohel D. (2024). Rvg: r graphics devices for 'Office' vector graphics output. Version 0.3.5.001. https://davidgohel.github.io/rvg/.

[16] Gosens T., Speigner B., Minekus J. (2009). Fracture of the scapular body: functional outcome after conservative treatment. J Shoulder Elbow Surg.

[17] Haddaway N.R., Grainger M.J., Gray C.T. (2021). Citationchaser: an R package and Shiny app for forward and backward citations chasing in academic searching. Zenodo.

[18] Hashiguchi H., Ito H. (2003). Clinical outcome of the treatment of floating shoulder by osteosynthesis for clavicular fracture alone. J Shoulder Elbow Surg.

[19] Heim K.A., Lantry J.M., Burke C.S., Roberts C.S. (2008). Early results of scapular fractures treated operatively at a level one trauma center. Eur J Trauma Emerg Surg.

[20] Hirt J., Nordhausen T., Fuerst T., Ewald H., Appenzeller-Herzog C., TARCiS study group (2024). Guidance on terminology, application, and reporting of citation searching: the TARCiS statement. BMJ.

[21] Izadpanah K., Jaeger M., Maier D., Kubosch D., Hammer T.O., Südkamp N.P. (2012). The floating shoulder—clinical and radiological results after intramedullary stabilization of the clavicle in cases with minor displacement of the scapular neck fracture. J Trauma Acute Care Surg.

[22] Jones C.B., Sietsema D.L. (2011). Analysis of operative versus nonoperative treatment of displaced scapular fractures. Clin Orthop Relat Res.

[23] Kim K.C., Rhee K.J., Shin H.D., Yang J.Y. (2008). Can the glenopolar angle be used to predict outcome and treatment of the floating shoulder?. J Trauma.

[24] Labler L., Platz A., Weishaupt D., Trentz O. (2004). Clinical and functional results after floating shoulder injuries. J Trauma.

[25] Lin T.L., Li Y.F., Hsu C.J., Hung C.H., Lin C.C., Fong Y.C. (2015). Clinical outcome and radiographic change of ipsilateral scapular neck and clavicular shaft fracture: comparison of operation and conservative treatment. J Orthop Surg Res.

[26] McGuinness L.A., Higgins J.P.T. (2021). Risk-of-bias VISualization (robvis): an R package and shiny web app for visualizing risk-of-bias assessments. Res Synth Methods.

[27] Mulawka B., Jacobson A.R., Schroder L.K., Cole P.A. (2015). Triple and quadruple disruptions of the superior shoulder suspensory complex. J Orthop Trauma.

[28] Page M.J., McKenzie J.E., Bossuyt P.M., Boutron I., Hoffmann T.C., Mulrow C.D. (2021). The PRISMA 2020 statement: an updated guideline for reporting systematic reviews. PLoS Med.

[29] R Core Team (2021).

[30] Rethlefsen M.L., Kirtley S., Waffenschmidt S., Ayala A.P., Moher D., Page M.J. (2021). PRISMA-S: an extension to the PRISMA statement for reporting literature searches in systematic reviews. J Med Libr Assoc.

[31] Rohit K., Jangir R., Verma V. (2023). Fractures of the scapula: results and functional outcomes of operative treatment. Int J Pharm Clin Res.

[32] Rollo G., Huri G., Meccariello L., Familiari F., Çetik R.M., Cataldi C. (2021). Scapular body fractures: short-term results of surgical management with extended indications. Injury.

[33] Samy M.A., Darwish A.E. (2017). Fixation of clavicle alone in floating shoulder injury: functional and radiological outcome. Acta Orthop Belg.

[34] Shao Y., Zhu X., Liu B., Ji C., Sun J., Chen G. (2023). Is fixation of both clavicle and scapula better than clavicle alone in surgical treatment of floating shoulder injury? A retrospective study. BMC Musculoskelet Disord.

[35] Sterne J.A.C., Hernán M.A., Reeves B.C., Savović J., Berkman N.D., Viswanathan M. (2016). ROBINS-I: a tool for assessing risk of bias in non-randomized studies of interventions. BMJ.

[36] van Noort A., te Slaa R.L., Marti R.K., van der Werken C. (2001). The floating shoulder: a multicentre study. J Bone Joint Surg Br.

[37] van Noort A., van Kampen A. (2005). Fractures of the scapula surgical neck: outcome after conservative treatment in 13 cases. Arch Orthop Trauma Surg.

[38] Vander Voort W., Wilkinson B., Bedard N., Hendrickson N., Willey M. (2022). The operative treatment of scapula fractures: an analysis of 10,097 patients. Iowa Orthop J.

[39] Veritas Health Innovation (2025).

[40] Zhang J., Li Y., Bi Y., Chu X., Cao Y. (2022). A comparative analysis of titanium anatomic plate and titanium reconstructive plate for treatment of extra-articular fractures of the scapula (Miller types IIb, IIc, and IV). J Shoulder Elbow Surg.

[41] Zhou Q., Chen B., Zho Y., Chen H., Wang Z., Liu J. (2017). Comparisons of shoulder function after treatment of floating shoulder injuries with different methods. Biomed Res.

[42] Zlowodzki M., Bhandari M., Zelle B.A., Kregor P.J., Cole P.A. (2006). Treatment of scapula fractures: systematic review of 520 fractures in 22 case series. J Orthop Trauma.

